# P-430. What Do We Talk About When We Talk About Neutropenic Enterocolitis in Children? a 194 Patient Retrospective Cohort from Mexico

**DOI:** 10.1093/ofid/ofaf695.646

**Published:** 2026-01-11

**Authors:** Benito E Michel Izeta, Almudena Laris Gonzalez

**Affiliations:** Hospital Infantil de Mexico Federico Gomez, Naucalpan, Mexico City, Mexico; Hospital Infantil de Mexico Federico Gomez, Naucalpan, Mexico City, Mexico

## Abstract

**Background:**

Neutropenic enterocolitis is a common diagnosis in pediatric oncology patients. Ultrasound is often used to measure colonic wall thickness as a diagnostic tool, but its value is uncertain. It is unclear whether a normal thickness can be safely used to rule out the diagnosis. Although it is not the gold standard for diagnosis, no studies have examined the outcomes of patients whose symptoms are compatible with neutropenic enterocolitis but who have normal colonic thickness.

**Methods:**

A retrospective cohort was studied using charts from a pediatric referral center in Mexico City over a ten-year period, from 2013 to 2023. A total of 194 patients were followed until discharge for a composite outcome including death, septic shock, PICU admission, or the need for emergent abdominal surgery. The relationship between colonic wall thickness and the outcome was explored.

**Results:**

Approximately 42% of patients had solid organ malignancy. The composite outcome occurred in 45% of patients with a thickened colon and in 26% of those without it. After adjusting for age, oncologic diagnosis, and neutrophil count, a thickened colon had an odds ratio (OR) of 2.11 (1.03–4.3) for adverse outcomes. The most common individual outcome by far was septic shock. Bloodstream infection was uncommon, occurring in 9% of patients.

**Conclusion:**

Pediatric patients diagnosed with neutropenic enterocolitis have a high risk of adverse outcomes even when no colonic wall thickening is present. A thickened colon wall is associated with double that risk.

**Disclosures:**

Almudena Laris Gonzalez, MD, MSc, Astra Zeneca: Advisor/Consultant|GSK: Honoraria|Sanofi: Advisor/Consultant

